# Non-equilibrium repressor binding kinetics link DNA damage dose to transcriptional timing within the SOS gene network

**DOI:** 10.1371/journal.pgen.1007405

**Published:** 2018-06-01

**Authors:** Matthew J. Culyba, Jeffrey M. Kubiak, Charlie Y. Mo, Mark Goulian, Rahul M. Kohli

**Affiliations:** 1 Department of Medicine, Division of Infectious Diseases, Perelman School of Medicine at the University of Pennsylvania, Philadelphia, PA, United States of America; 2 Department of Biology, University of Pennsylvania, Philadelphia, PA, United States of America; 3 Department of Biochemistry and Biophysics, University of Pennsylvania, Philadelphia, PA, United States of America; Université Paris Descartes, INSERM U1001, FRANCE

## Abstract

Biochemical pathways are often genetically encoded as simple transcription regulation networks, where one transcription factor regulates the expression of multiple genes in a pathway. The relative timing of each promoter’s activation and shut-off within the network can impact physiology. In the DNA damage repair pathway (known as the SOS response) of *Escherichia coli*, approximately 40 genes are regulated by the LexA repressor. After a DNA damaging event, LexA degradation triggers SOS gene transcription, which is temporally separated into subsets of ‘early’, ‘middle’, and ‘late’ genes. Although this feature plays an important role in regulating the SOS response, both the range of this separation and its underlying mechanism are not experimentally defined. Here we show that, at low doses of DNA damage, the timing of promoter activities is not separated. Instead, timing differences only emerge at higher levels of DNA damage and increase as a function of DNA damage dose. To understand mechanism, we derived a series of synthetic SOS gene promoters which vary in LexA-operator binding kinetics, but are otherwise identical, and then studied their activity over a large dose-range of DNA damage. In distinction to established models based on rapid equilibrium assumptions, the data best fit a kinetic model of repressor occupancy at promoters, where the drop in cellular LexA levels associated with higher doses of DNA damage leads to *non-equilibrium* binding kinetics of LexA at operators. Operators with slow LexA binding kinetics achieve their minimal occupancy state at later times than operators with fast binding kinetics, resulting in a time separation of peak promoter activity between genes. These data provide insight into this remarkable feature of the SOS pathway by demonstrating how a single transcription factor can be employed to control the relative timing of each gene’s transcription as a function of stimulus dose.

## Author summary

As the precise timing of gene expression is critical for cells to respond and adapt to new environments, it is important to understand the underlying mechanisms which control this timing. In this report, we studied the timing of transcription for genes in the bacterial DNA damage repair pathway (known as the SOS response), a regulatory system where each gene is controlled by the same transcriptional repressor, LexA. By specifically isolating the role of the LexA binding interaction at SOS gene promoters, we found a relationship between the amount of DNA damage incurred by the cell, LexA binding kinetics at a promoter, and the timing of promoter activation. Our data fit a kinetic model that reveals how a disequilibrium between the LexA-operator binding reaction and cellular LexA concentrations causes timing differences between genes to emerge only at higher doses of DNA damage. Taken together, we show that *non-equilibrium* DNA binding kinetics is the mechanism by which a single transcription factor can modulate timing differences across an entire network of genes as a function of stimulus dose.

## Introduction

Transcription regulation networks enable a cell to exert exquisite control over its biochemical pathways and are prevalent across the tree of life [1]. The gene promoters in simple network motifs share a single common transcription factor, thus enabling expression of an entire set of pathway genes in response to a specific environmental stimulus. The timing of promoter activation and shut-off for each gene in a network can be significantly different, which can affect pathway dynamics and physiology, thus making it an important goal to understand how the relative timing of promoter activities is achieved. For example, the flagellar biosynthetic pathway in *Escherichia coli* follows a sequence corresponding to the spatial positioning of the gene products in the assembled flagellar motor, going from the cytoplasmic to the extracellular sides, and ending with the chemotaxis navigation system and components needed for motor torque generation [2]. Similarly, the gene networks encoding sets of amino acid biosynthetic enzymes have been shown to be transcribed in the same order they are needed in their biochemical pathway. This phenomenon, termed just-in-time transcription, likely ensures that energy is not expended on expressing genes before their products can be fully actualized in the pathway [3]. The timing of gene transcription is also believed to be a prominent feature of the DNA damage repair pathway in bacteria (known as the SOS response). In the SOS response, however, the sequential timing of gene induction is believed to facilitate a transition from high-fidelity DNA repair early in the response to low-fidelity (i.e. mutagenic) DNA repair activities late in the response [4]. Once DNA repair is complete the response begins to shut off; therefore, the more expeditious timing of high-fidelity repair may serve to restrict the mutagenic and toxic activities of late genes only to settings where the high-fidelity systems are overwhelmed. It is hypothesized that such a balance may tune the mutation rate to the level of environmental stress, since the fitness cost of deleterious mutations can be outweighed by the reward of a less-probable beneficial ‘escape mutation’ that ameliorates the stress [5, 6]. Another remarkable aspect of the SOS pathway is that, unlike other pathways that exhibit temporal ordering of gene transcription [2, 7], the temporal ordering of SOS genes is not attributable to a serial cascade of transcription factors, but instead appears to involve only one repressor protein.

**Fig 1:**
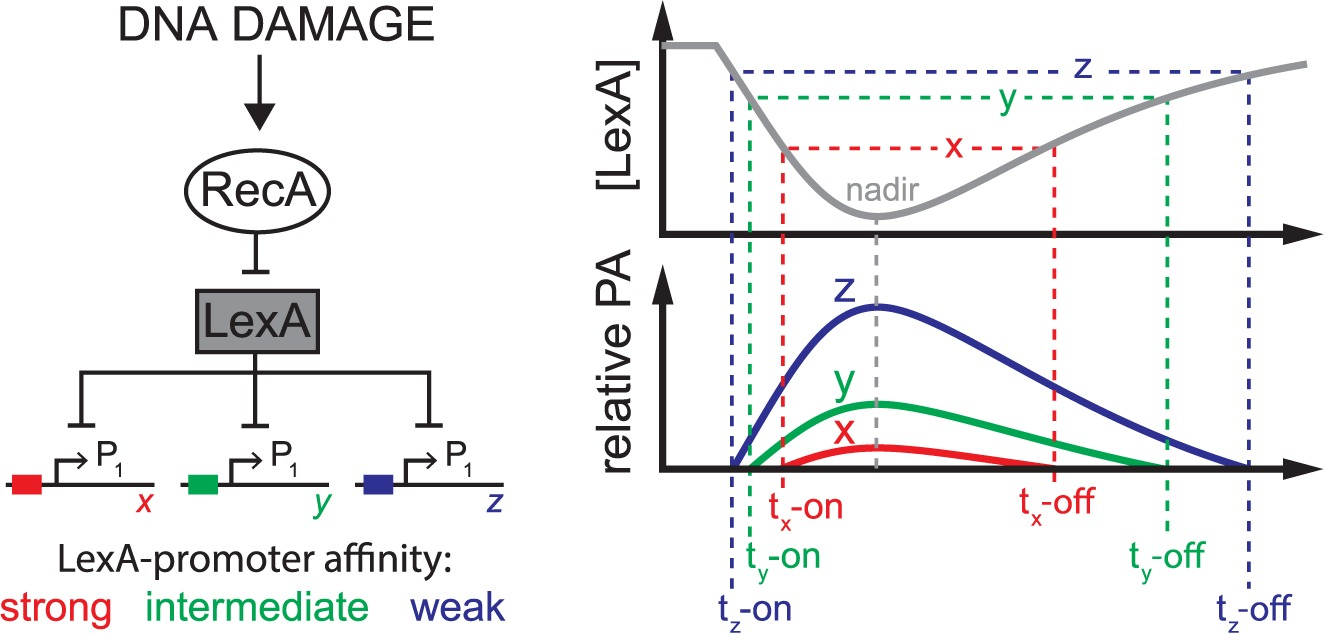
Thermodynamic equilibrium model of LexA occupancy at SOS promoters. Left: RecA/LexA regulation of the SOS gene network in response to DNA damage. LexA represses transcription at hypothetical SOS gene promoters, x, y, and z, of similar promoter strengths, but with relatively strong (red), intermediate (green), or weak (blue) binding affinity for LexA. Right: After DNA damage, the LexA concentration (gray line) falls, reaches a nadir, then re-accumulates after DNA repair. In this model, the LexA-promoter binding reaction is in thermodynamic equilibrium with falling and rising LexA levels, therefore, the relative promoter activities of x, y, and z are dependent on promoter binding affinity for LexA, as defined by the equilibrium binding constant, K_d_. Horizontal dotted lines indicate threshold concentrations of LexA at which the promoters achieve the identical promoter activity level (arbitrarily defined here as the y-intercept value of the x-axis). As LexA levels fall or rise, the time at which this promoter activity level is reached, t-on or t-off, respectively, is different for each promoter and indicated by the colored vertical dotted lines. The grey dotted line indicates the time of the LexA nadir. Given conditions of thermodynamic equilibrium for LexA binding at the promoters, the model predicts the time of peak promoter activity will correspond to the time of the LexA nadir for all promoters and that the first promoters to turn on will be the last to turn off (first-on last-off pattern). The limitations of this equilibrium model are discussed in the text and stand in contrast to the kinetic model (see Fig 7).

The SOS pathway serves as a robust model system to study timing, since the basic regulatory features of the response are known [8]. In *E*. *coli*, about 40 SOS-regulated genes are induced following a genotoxic insult [9]. Each gene in the SOS pathway is regulated by the LexA repressor. LexA represses SOS genes by specifically binding to operator DNA sequences within promoters and inhibiting transcription. Operator sequences are 20 base pair imperfect palindromes consisting of two functional half-sites. LexA binds to operator DNA as a dimer, with each operator half-site engaged by one LexA monomer. Operators require two conserved 5’-CTG-3’ motifs spaced precisely ten base pairs apart to support specific binding by LexA, but the remaining sequence is variable between operators, imparting different LexA binding affinities for each [10]. Most SOS genes contain a single operator, although the number and location of operators relative to the transcription start site can vary [11]. In the absence of DNA damage, the cell maintains LexA at high concentrations, LexA is bound to operators, and SOS promoters are repressed. In the setting of DNA damage, however, LexA is triggered to undergo a self-cleavage reaction mediated by its protease domain, which results in a loss of its DNA binding and repressor activities. The DNA damage signal is transduced to LexA by the RecA protein [12]. This occurs after DNA damage leads to the exposure of single-stranded DNA (ssDNA) at stalled replication forks. RecA turns into its active form (termed RecA*) by binding to and polymerizing along the ssDNA, and it is this RecA* form that serves as the co-protease that stimulates the LexA self-cleavage reaction [8]. The subsequent depletion of cellular LexA levels marks the start of the SOS response (Fig 1). The concentration of LexA decreases over a time-scale of several minutes, reaches a nadir, and then re-accumulates after repair is achieved. Notably, greater amounts of DNA damage lead to lower concentrations of LexA and slower recovery to steady-state levels [13].

The effect a transcriptional repressor (or activator) has on promoter activity is often modeled assuming that the repressor-promoter binding reaction is in thermodynamic equilibrium with dynamic repressor levels inside the cell. For the SOS gene network, the LexA repressor serves as the master transcriptional regulator, and the temporal dynamics of LexA depletion and re-accumulation within the cell will, in part, dictate LexA occupancy at SOS gene promoters. The ‘equilibrium model’ posits that the kinetics of LexA interactions at promoters are sufficiently fast to be in thermodynamic equilibrium with LexA depletion and re-accumulation dynamics [14]. In this model, the cellular concentration of active LexA repressor determines the LexA occupancy state of the promoter and thus the timing of promoter activation. Weaker LexA-affinity promoters are expected to activate at earlier times (higher LexA concentration thresholds for activation) relative to stronger LexA-affinity promoters [15] and, by the same logic, the weaker affinity promoters are expected to shut-off at relatively later times than stronger affinity promoters (Fig 1). Sequential timing of promoter activation in the SOS network has been indirectly inferred via this model; this inference is derived from biochemical measurements showing that the equilibrium binding affinity of LexA is different across SOS gene promoters, which is presumed to significantly affect the timing of promoter activation between genes [16]. The equilibrium model provides an explanation for how promoters may turn on and off at different times and, importantly, provides testable predictions. First, the model predicts that all SOS promoters will display peak activity at the same time, since peak activity will coincide with the LexA nadir for all genes, and second, it predicts that that temporal ordering will follow a ‘first-on last-off’ pattern according to the equilibrium binding affinities of the repressor for each promoter (Fig 1).

In contrast to the equilibrium model predictions, direct measurements of SOS promoter activity showed promoters displaying peak activity at different times [15]. However, the mechanism which governs the distinct timing of different genes is unclear. In biochemical experiments with purified components, dissociation of LexA from some operators occurs on a time-scale of minutes, raising the possibility that slow dissociation kinetics could cause a delay in transcription for some SOS genes due to ‘non-equilibrium’ LexA binding dynamics [17]. However, there are also a myriad of additional promoter features which could affect the timing of a subset of SOS genes, such as additional transcription factors [18, 19], promoter strength [3], the number and positions of operators relative to the transcription start site [11], or the kinetics of the RNA polymerase (RNAP)-promoter interaction [20]. Given that these additional factors confound comparison between SOS genes, it remains uncertain as to whether the equilibrium model of LexA promoter occupancy is correct or if, instead, non-equilibrium dynamics are involved [21].

To address these deficits, we devised a system utilizing green fluorescent protein (GFP) transcription-reporters that could specifically assess how the LexA-operator binding reaction influences the kinetics of promoter activity. First, using GFP-reporter plasmids, we measured promoter activity kinetics of native *E*. *coli* SOS genes and found a correlation between the LexA-operator dissociation rate and the timing of peak promoter activity, suggesting the LexA-operator interaction plays a significant role in the timing of the SOS response. Then, to establish causality, we used just one reporter plasmid as a common promoter template to construct a series of synthetic SOS promoters with different LexA-operator dissociation rates, thus removing the confounding effects of comparing promoters from different genes. Additionally, to control for plasmid artifacts, we created *E*. *coli* strains with GFP-reporter cassettes engineered into the chromosome as single copies. In both the plasmid- and chromosome-based systems, we found the LexA-operator interaction alone plays a large role in determining the wide range of peak promoter activity times observed. Interestingly, the level of DNA damage affected timing differently for each synthetic promoter and these differences depended on the rate of LexA binding kinetics at operators. Finally, we derived a kinetic model of LexA occupancy at SOS gene promoters which explain these data, thus providing a quantitative framework for how a single transcription factor can be employed to modulate timing differences across an entire network of genes as a function of stimulus dose.

## Results

### Measurement of promoter activity kinetics

Prior studies of SOS gene transcription have either studied relatively few genes [15], lacked the temporal resolution or sensitivity to accurately assign promoter activity peaks [9], and/or induced DNA damage over a narrow dose-range [9, 15]. We hypothesized that SOS gene promoter activity patterns would show a high degree of variation when studied over a large dose-range of DNA damage, therefore, we first sought to better characterize the full range of promoter activity patterns. To do this, we employed a previously described GFP transcription-reporter method [22] to monitor the promoter activity of fourteen *E*. *coli* SOS genes [9] through time over a wide dose-range (0.2–100 J/m^2^) of ultraviolet light (UV)-induced DNA damage. In this method, the gene encoding a fast-folding, long-lived GFP variant (GFPmut2) is placed under control of the promoter of interest in a very low copy number plasmid (S1A Fig), thus enabling quantitative measurements of GFP fluorescence in live cells with high temporal resolution [22]. The rate of mRNA synthesis from the promoter (herein referred to as ‘promoter activity’) is then inferred from the change in the GFP fluorescence signal over time (see Materials and Methods). To ensure the GFP signal was dependent on DNA damage, was specific to SOS gene promoters, and relied on LexA self-cleavage activity, we carried out control experiments with no UV exposure, a GFP-reporter plasmid for the non-SOS *lac* promoter, and a bacterial strain which encodes a LexA-variant (*lexA*^S119A^) incapable of self-cleavage, respectively. As anticipated, we found no detectable GFP signal in these control experiments (S1B Fig).

### DNA damage dose-dependence of promoter activity kinetics

**Fig 2:**
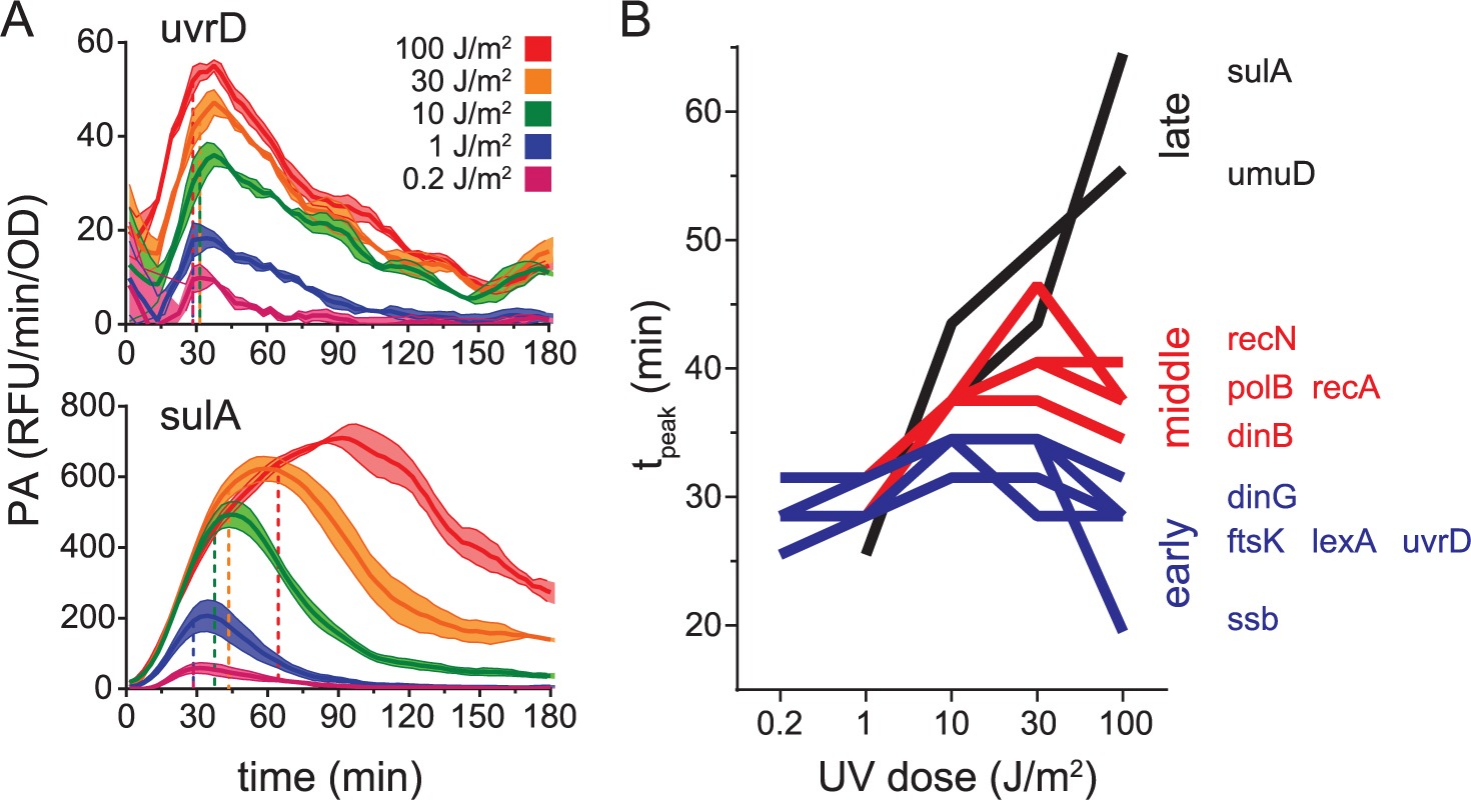
Effect of UV dose on the timing of peak promoter activity. A. Promoter activity for the *uvrD* and *sulA* promoters plotted as a function of time after a range of UV doses. Promoter activity (PA) measurements were acquired at 3 minute intervals. Solid lines and shading of the same color connect data points and indicate the mean and standard error of promoter activity measurements, respectively. Colors indicate UV dose, as shown in the figure legend. Horizontal dotted lines indicate the value of 90% of peak promoter activity (0.9*PA_peak_) for that trace and vertical dotted lines indicate the time at which 0.9*PA_peak_ occurred (t_peak_). B. t_peak_ plotted as a function of UV dose. Promoters were binned into early (blue), middle (red), or late (black) time categories based on t_peak_ values at 100 J/m^2^. Lines connecting data points from each UV dose are shown for ease of visualization.

The temporal promoter activity patterns of the fourteen genes varied considerably as a function of UV dose. The UV dose led to changes in both the extent of promoter activity and the timing of peak activity. We first focused our attention on the trends notable in the relative timing of peak activity. At lower UV doses (≤ 1 J/m^2^), the time at which peak promoter activity occurred was similar between genes and normalization of the activity traces by peak promoter activity revealed nearly superimposable activity plots (S2 Fig). At higher UV doses (≥ 10 J/m^2^), however, the traces diverged from one another and the time at which peak promoter activity occurred was different between genes (S2 Fig). Additionally, three promoters appeared to be outliers. For *sbmC* and *ruvA*, we observed a strong biphasic response in temporal promoter activity, with a larger second peak occurring at 120 min and 165 min, respectively, at the highest UV dose (S2 Fig). Of note, *sbmC* has been reported previously as a stationary-phase induced SOS gene [23]. In contrast, we noted *ybfE* was strongly downregulated at late times compared to the other genes (S2 Fig). Unlike the other eleven promoters, which are predicted to utilize the ‘house-keeping’ sigma-factor, RpoD (σ^70^), the same prediction tool does not predict σ^70^ utilization for *sbmC*, *ruvA*, or *ybfE* [24]. Suspecting alternative sigma-factor utilization, we elected to exclude these three genes from subsequent analysis on this basis. However, even amongst the remaining eleven genes, the amount of DNA damage incurred by the cell significantly changed the relative timing of promoter activity. For example, *uvrD* promoter activity peaked between 35–38 min at all the UV doses studied, but *sulA* promoter activity peaked at progressively later times as the UV dose was increased, reaching a peak activity at 92 min at the highest dose studied (Fig 2A).

To facilitate quantitative comparison between gene promoters, we defined PA_peak_ as the highest value of promoter activity observed in the trace and t_peak_ as the amount of time which elapsed between UV exposure and 90% of the PA_peak_ value, then applied these definitions to all the promoter activity traces (Figs 2A and S3). The 90% PA_peak_ threshold was chosen to eliminate miscalling the true t_peak_ due to increased measurement error surrounding promoter activity peaks. However, with this definition, we find the error for t_peak_ values is less than the period between the measurement intervals, and so we conservatively estimate the error associated with t_peak_ values to be ±3 min. Our analysis revealed that, at low UV doses (≤ 1 J/m^2^), t_peak_ values were similar between all SOS gene promoters, ranging from 25–32 min, but at higher UV doses (≥ 10 J/m^2^) t_peak_ values varied widely across SOS genes, ranging from 19–65 min (Fig 2B). We noted that *dinG*, *ftsK*, *lexA*, and *uvrD* did not display significant alterations in t_peak_ over the wide range of UV doses studied and *ssb* achieved t_peak_ at an earlier time at the highest UV dose. In contrast, the remaining promoters exhibited t_peak_ values which increased in a UV dose-dependent manner. For example, *dinB*, *polB*, *recA*, and *recN* displayed a moderate increase in t_peak_, achieving values of 34–47 min at higher UV doses, and *sulA* and *umuD* exhibited markedly increased values of 56 and 65 min, respectively, at the highest dose studied. These results agree well with a prior study that noted timing differences in peak promoter activity after a UV dose of 20 J/m^2^ [15], however, our extended analysis reveals a new feature: The timing differences between genes are absent at lower UV doses and are exacerbated even further at higher UV doses. This occurs because the timing of peak promoter activity changes differently for each promoter as a function of UV dose.

The magnitude of promoter activity also varied significantly between genes. Nine of the genes displayed values of <100 relative fluorescence units per minute per optical density (RFU/min/OD) for their highest PA_peak_, four had intermediate values of 100–1000 RFU/min/OD, and one gene, *recA*, had the largest observed value of ~1700 RFU/min/OD (S3 Fig). To facilitate quantitative comparison of the magnitude of promoter activity between the SOS genes across the entire range of DNA damage imposed, we used a three-parameter dose-response curve (see Eq 6 in Materials and Methods) to fit the PA_peak_ data (R^2^ range = 0.83–0.99). This enabled estimates of the effective dose of UV corresponding to half-maximal activation of the promoter (ED_50_) and the maximal promoter activity (PA_max_) for each promoter (S4A Fig and S1 Table). ED_50_ values varied from 0.4 to 11 J/m^2^ and we noted that some promoters, such as that of *dinG* and *lexA*, had relatively low ED_50_ values and achieved PA_max_ at doses of <10 J/m^2^, suggesting the promoter is completely unoccupied even at this relatively low dose. In contrast, other promoters, such as that of *sulA* and *umuD*, displayed relatively high ED_50_ values and required the highest dose of 100 J/m^2^ to achieve PA_max_ (S4A Fig). This analysis agrees with past studies of the SOS regulon which demonstrate that, at intermediate levels of LexA repression, some promoters are highly induced, whereas others are only partially induced [25]. Given these results, it seemed plausible that PA_max_ values corresponded to the promoter activity level of a completely LexA-unoccupied promoter state. To gain further insight in the meaning of these model parameters, we measured basal GFP levels in the absence of DNA damage in both Δ*lexA* and *lexA*^+^ cells to capture the extremes of LexA de-repression and repression, respectively. Then, we compared these data to the PA_max_ values we obtained from the dose-response model. We found the values of PA_max_ were highly correlated with basal GFP levels in Δ*lexA* cells (r = 1.00, P< 0.0001), but not *lexA*^+^ cells (r = 0.15, P = 0.66), providing further evidence that the model parameter PA_max_ represents the activity of the LexA-unoccupied promoter inside cells (S4B Fig). Our analysis thus demonstrates that maximal promoter activities (PA_max_ values) induced by DNA damage differ based on the promoter activity of the LexA-unoccupied state, and that promoter activation thresholds (ED_50_ values) are independent of promoter strength.

### LexA-operator dissociation rate correlates with promoter activity kinetics

Having quantified promoter activity of a large set of SOS gene via two parameters which describe the timing (t_peak_) and the UV dose activation threshold (ED_50_) for each promoter, we next investigated whether these parameters might correlate to specific SOS promoter features that also vary across SOS genes. We found no correlations of these parameters with the number or location (relative to the transcription start site) of LexA operator sequences or to the PA_max_ values (promoter strength) we determined for each promoter (S1 Table).

**Fig 3:**
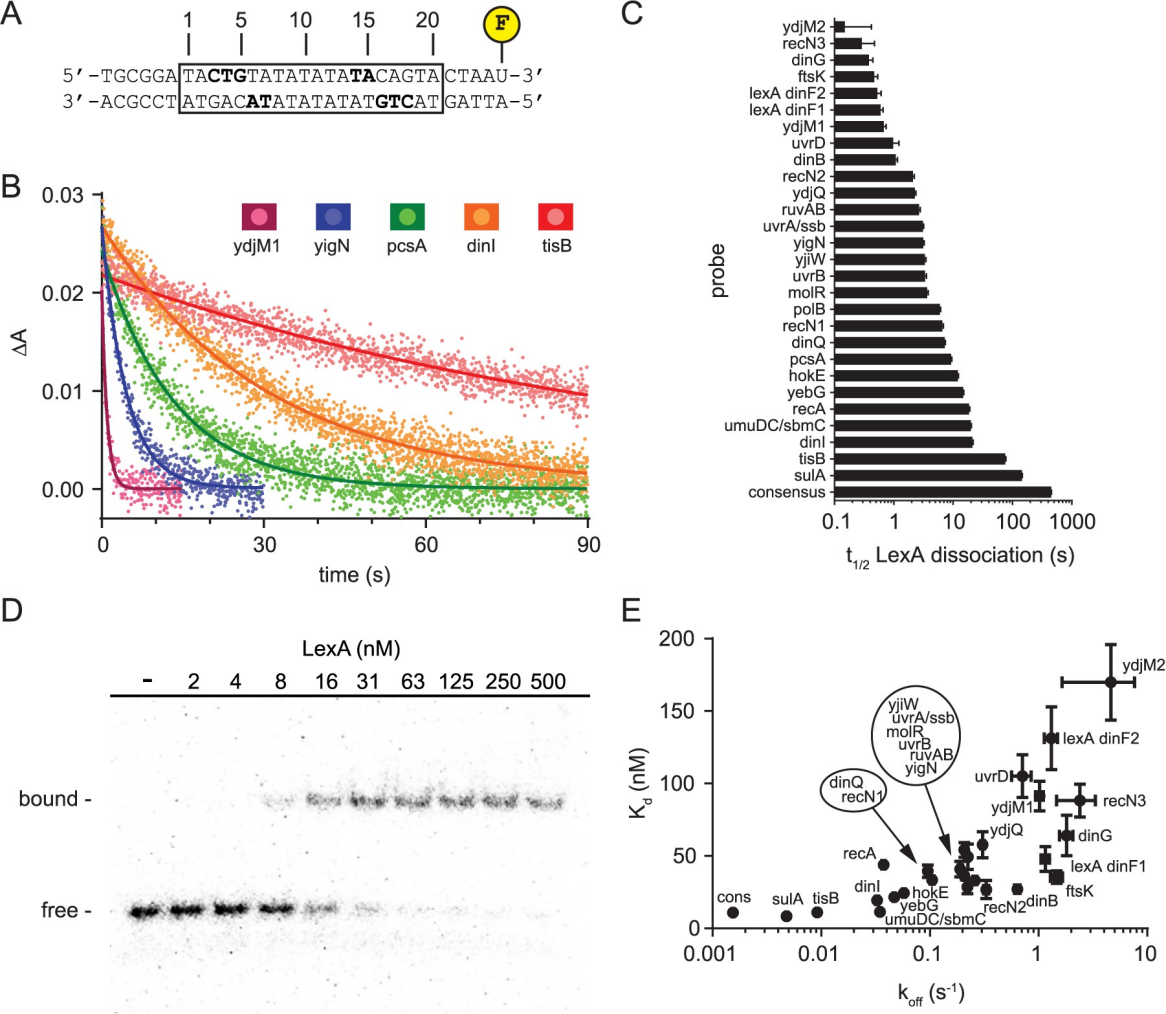
LexA binding to native *E*. *coli* operators. A. Design of FAM-labeled dsDNA probes for fluorescence anisotropy-based assay to measure LexA dissociation from operator DNA. The boxed region indicates the 20 base pair operator sequence implicated in LexA binding, which was different for every probe. The flanking DNA sequence was kept constant between probes. Bolded residues indicate residues with specific nucleobase contacts to LexA [10]. B. Dissociation curves for representative operators from rapid mixing of a preformed LexA-DNA complex with excess unlabeled operator DNA. Data points and solid lines of the same color indicate the mean anisotropy values at each time point and the best-fit curve for a simple exponential decay model, respectively. C. t_1/2_ values for all the *E*. *coli* LexA operators included in this study arranged by t_1/2_. Error bars represent 95% confidence intervals derived from non-linear regression. D. Representative gel image from EMSA analysis. EMSA probes are the same as in 1A except ^32^P-labeled instead of FAM-labeled. Data for the *dinI* operator are shown. E. Plot of apparent K_d_ versus k_off_ for the LexA-operator dissociation reaction. K_d_ values were determined by EMSA and k_off_ values were determined by the fluorescence anisotropy-based dissociation assay. Error bars represent 95% confidence intervals of parameter estimates derived from non-linear regression (see Materials and Methods). Data points are labeled with SOS gene names. Due to space constraints, the placement of some data labels is offset. To enable association of each offset label with its proper data point, these labels are circled and the relative spatial orientation of the names within the circle reflects the same orientation of the data points they represent, whose locations are indicated by the arrows.

Next, to understand if the variation in t_peak_ and ED_50_ for the promoters could, in part, be attributed to the biochemical parameters that describe the LexA-operator interaction, we measured both the LexA dissociation rates and the equilibrium binding constants for 28 unique operator DNA sequences found within the *E*. coli chromosome. Although the effect a repressor (or activator) has on promoter activity is often modeled using its equilibrium binding affinity (K_d_) for the promoter, the kinetic parameters (k_off_, k_on_) are of interest, because if LexA binding at operators is not in thermodynamic equilibrium with falling and rising LexA levels within cells, then differences in the timing of promoter activation could be attributed to differences in LexA off-rates and on-rates. To quantify dissociation rates, we developed an approach based on fluorescence anisotropy (Fig 3A). Fluorescently labeled double-stranded DNA (dsDNA) probes were constructed, each containing a unique operator sequence. Then, for each probe, pre-formed LexA-DNA complex was rapidly mixed with a vast excess of unlabeled operator DNA using a stopped-flow instrument and the change in anisotropy associated with LexA dissociation from the labeled complex was measured through time. The data were fit to a simple exponential decay model yielding the half-life (t_1/2_) of the dissociation reaction (Fig 3B), which is inversely proportional to the apparent rate constant of the reaction, k_off_ (t_1/2_ = ln2/k_off_). Larger t_1/2_ values (or smaller k_off_ values) indicate slower dissociation. We found t_1/2_ values spanned a wide range of ~2.5 logs (Fig 3C).

The N-terminal domain of the LexA repressor contains its DNA binding activity. The recombinant version of LexA used above contains an N-terminal hexahistidine tag used for affinity purification, which could potentially interfere with measurements of DNA binding. Therefore, as an additional control, we removed the affinity tag for comparison and found the relative LexA dissociation rates between different operators was unchanged (S5 Fig).

Next, we measured the LexA equilibrium binding constants (K_d_) for the same probes using an electromobility shift assay (EMSA) (Fig 3D). We found the apparent K_d_ values correlated to k_off_ values (r = 0.74, P< 0.0001), but spanned only a 20-fold range (8–170 nM) and were especially poor at distinguishing between operators with smaller k_off_ values (Fig 3E). Given the relationship K_d_ = k_off_/k_on_, these data indicated that the inferred k_on_ values show a positive correlation to k_off_ values, but must vary over a relatively smaller range. Given the importance of understanding how these parameters co-vary across different operators in order to inform our kinetic model (discussed further below), we measured both the off-rates and on-rates for a set of sixteen different LexA operators using the fluorescence anisotropy assay. Consistent with the above prediction, we found k_on_ values positively correlated with k_off_ values (Pearson r = 0.98, P< 0.0001), but displayed a much smaller range of values (S6 Fig). We conclude that, across a large set of operators, k_off_, k_on_, and K_d_ values are positively correlated, but k_off_ is the biochemical parameter that most strongly discriminates between LexA-operator binding interactions at different operators. As such, we elected to use the dissociation rates alone to perform subsequent correlations with the promoter activity parameters t_peak_ and ED_50_.

To understand if LexA-operator dissociation kinetics could in part explain the range of ED_50_ and t_peak_ values from the promoters we studied, we used the subset of measured t_1/2_ values which corresponded to these promoters to test for correlations. We found the biochemically determined t_1/2_ values significantly correlated with both ED_50_ (r = 0.80, P = 0.005) and the t_peak_ values obtained at 100 J/m^2^ (r = 0.78 and P< 0.01) (S1 Table). However, given the myriad of possible LexA-independent confounding factors mentioned above, these correlations only suggested, but did not prove, a causal mechanism involving the LexA-operator interaction (discussed further below).

### Construction and validation of *recA* promoters engineered with different LexA operators

**Fig 4:**
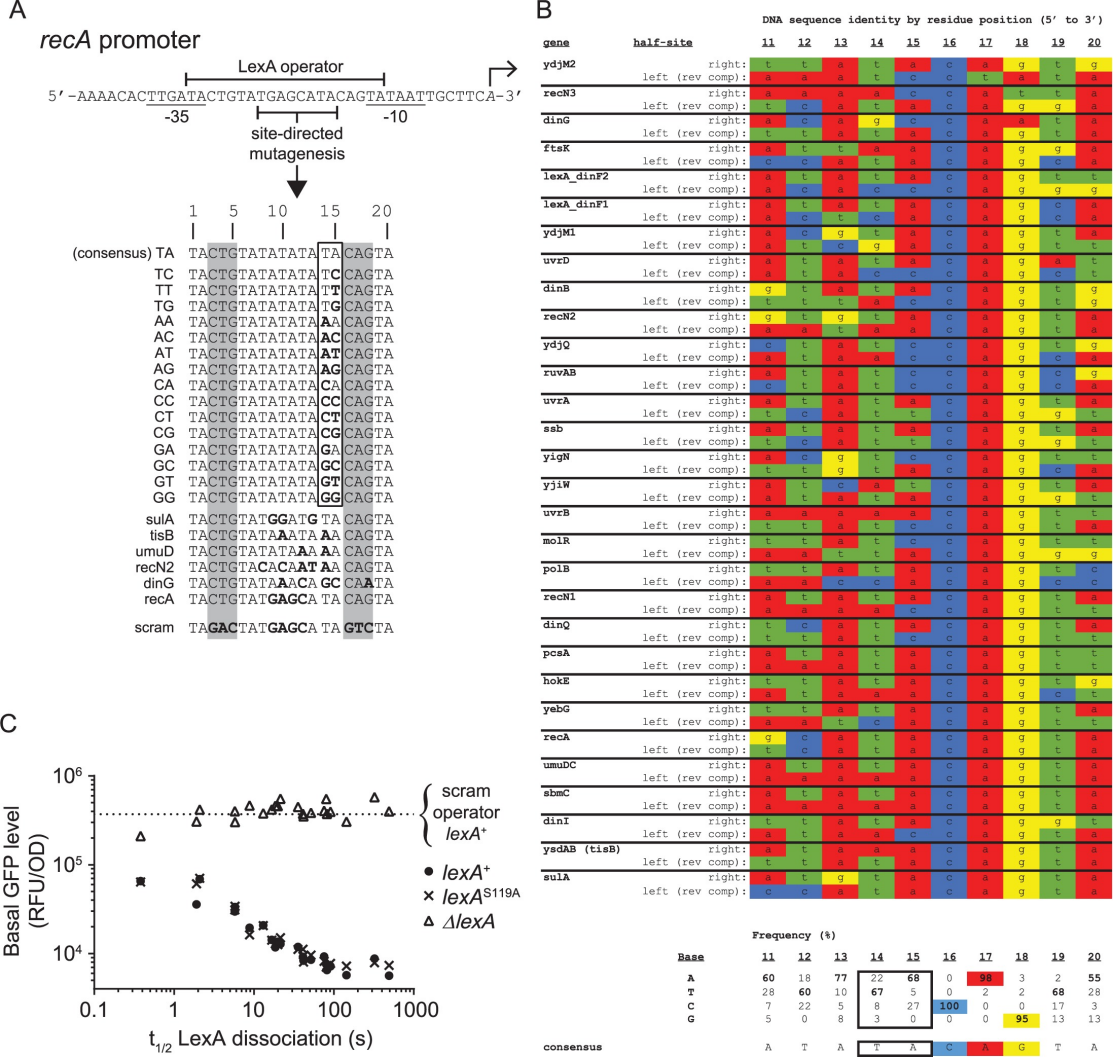
Construction and validation of *recA* promoters engineered with different LexA operators. A. Site-directed mutagenesis of the *recA* promoter’s operator sequence to create 22 synthetic SOS promoters. Transcription start site is indicated by rightward facing arrow and conserved -10 and -35 RNAP binding sites are underlined. LexA operator sequence indicated by top bracket. Mutagenesis was restricted to the region indicated, except for base pair position 18 of the *dinG* operator. The first group of sixteen promoters was engineered to contain operator sequences which mimic the consensus operator and are identical to one another, except for operator base pair positions 14 and 15. The second group of six promoters was engineered to contain operator sequences found in *E*. *coli* SOS genes. Bolded residues indicate deviations from the consensus operator sequence. The ‘scram’ promoter contains an operator sequence in which the highly conserved CTG (CAG)-motifs of the LexA operator sequence (grey shading) were mutated to ablate LexA binding. Binding is not detectable to the scram operator in biochemical assays at the highest LexA concentration tested of 1 μM. B. LexA operator half-site alignment. LexA binds to its 20 bp operator DNA as a dimer. Operators are comprised of two half-sites, which exhibit dyad symmetry with respect to highly conserved CTG (CAG)-motifs. Each monomer of a LexA dimer engages one half-site of the operator. The DNA sequence alignment contains 60 half-sites, derived from 30 operators in the *E*. *coli* chromosome. Sequences are arranged as in Fig 3C, in order of increasing t_1/2_ value. For each operator, the DNA sequence of the ‘right’ half-site is shown above the ‘left’ half-site. ‘Left’ half-site sequences are reverse complemented to account for the dyad symmetry. Residue frequencies for each position and the consensus half-site sequence are given below the alignment. The consensus frequency values are bolded, the highly conserved CTG (CAG)-motif is highlighted, and the residues targeted for mutagenesis in A (positions 14 and 15) are outlined with a black border. C. Basal promoter activity of the synthetic *recA* promoters as a function of LexA-operator dissociation rate (t_1/2_) in Δ*lexA*, *lexA*^+^, and *lexA*^S119A^ cells. Horizontal dotted line indicates the value obtained with the ‘scram’ control promoter in the *lexA*^+^ strain.

Given the complexity of comparing native *E*. *coli* promoters to one another noted above, we sought a system that would isolate the ‘strength’ of the LexA-operator interaction as the only variable between promoters. We reasoned this goal could be accomplished by making a series of synthetic promoters, based on a common promoter template, which differed only in the LexA operator sequence. We elected to use the promoter from the canonical SOS gene, *recA*, as the template for this series since this promoter exhibited the most robust signal of the reporter plasmids we studied. Also, the operator sequence is located upstream of the transcription start site and directly between the -10 and -35 RNA polymerase binding sequences (Fig 4A). These latter features allow for a consistent mRNA product between the plasmids in the series and enabled us to change the internal portions of the operator sequence without altering the conserved portions of the RNA polymerase binding site. Nevertheless, engineering the *recA* promoter to not only span the full range of the native *E*. *coli* LexA-operator dissociation rates we observed, but also to do so with minimal perturbation of promoter function, posed a significant design challenge.

To overcome this challenge, we rationally designed a series of synthetic operators which differed at only two base pairs at an internal location within the operator sequence. First, we examined the previously determined crystal structure of a LexA-operator complex [10] and a DNA sequence alignment of *E*. *coli* LexA operator half-sites (Fig 4B). In the crystal structure, half-site residues 14–18 make specific nucleobase contacts with LexA. However, in the sequence alignment, the identity of residues 16–18 (CAG) is virtually invariant, while the identity of residues 14 and 15 is variable (Fig 4B). As expected from the alignment, base substitution at residues 16–18 is known to severely abrogate LexA binding [11], therefore altering these positions would not allow us to create the wide range of LexA binding strengths we desired. In contrast, the variability present at residues 14 and 15 suggested base substitutions at these positions would still allow for productive LexA-operator complex formation, but alter the kinetics of the binding interaction, which in turn, may translate to differential promoter activities in cells. Although the DNA sequences and dissociation rates of the 28 native *E*. *coli* operators we measured revealed no obvious correlations between the identity of residues 14 and 15 and the dissociation rates (Fig 4B), we suspected this apparent lack of a correlation was confounded by co-varying sequence diversity present at other positions in these operators. Therefore, we reasoned that making substitutions at operator residues 14 and 15 on an otherwise identical operator sequence would unmask the contribution of these residues to the interaction with LexA.

**Fig 5:**
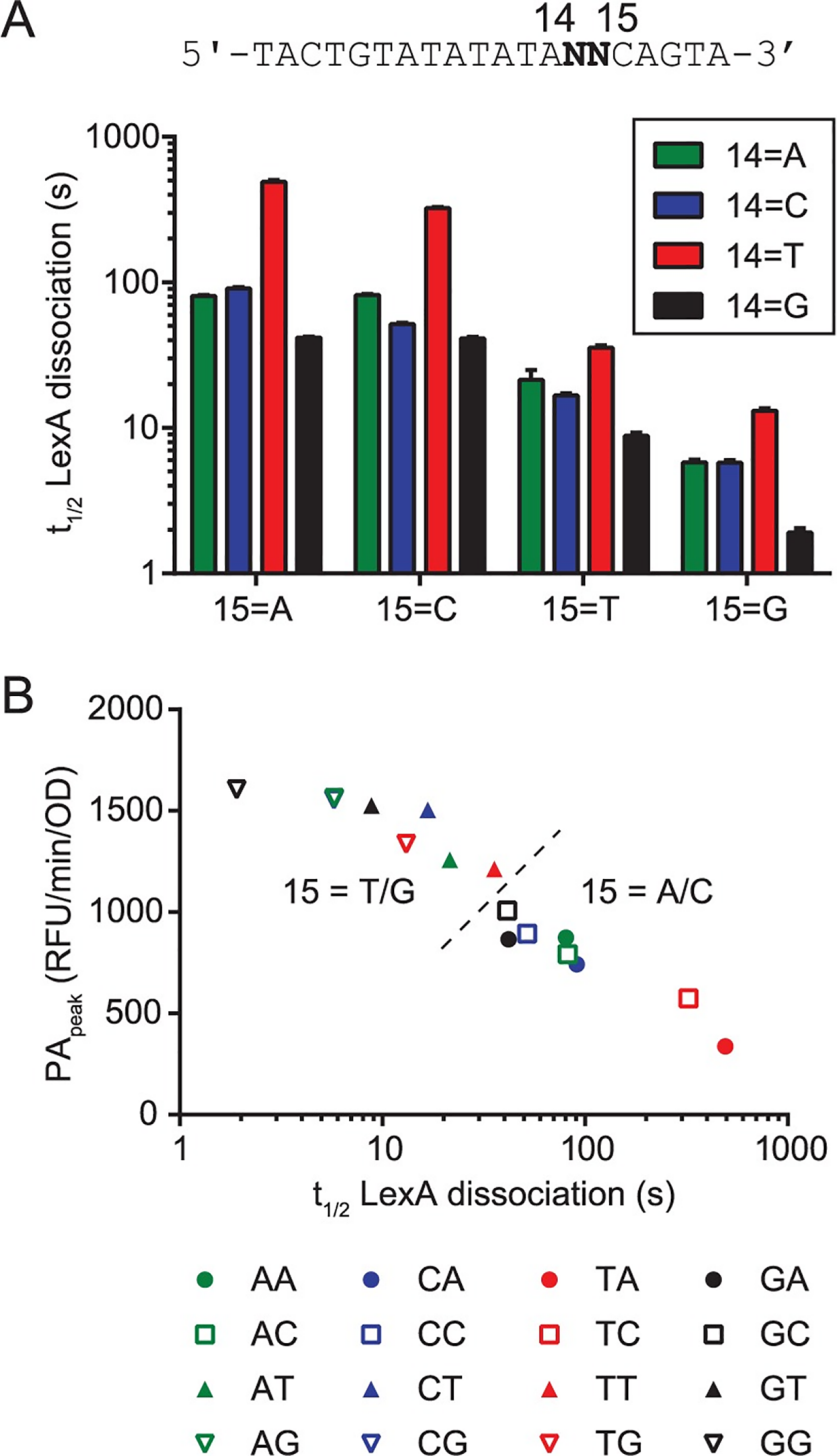
Analysis of operator residues 14 and 15 on LexA binding and promoter activity. A. LexA-operator dissociation rates (t_1/2_) for all sixteen possible operators containing Watson-Crick base pair substitutions at residues 14 and 15. Data are arranged according to residue position, where the x-axis labels indicate the identity of residue 15 and colors indicate the identity of residue 14 (see legend). The core DNA sequence of the operator probes is shown above the plot, where N indicates the location of the residues which vary between the sixteen different operators. B. Values of PA_peak_ were obtained after a UV dose of 10 J/m^2^ and are derived from the set of sixteen GFP-reporter plasmids containing the same operator sequences as in A, and are plotted against the biochemically determined t_1/2_ values for the LexA-operator dissociation reaction. Legend: Two-letter designations refer to the operator DNA sequences in Fig 4A. Different colors indicate the identity of residue 14 and different symbols indicate the identity of residue 15. The dashed line indicates separation of the dataset into two groups (15 = T/G and 15 = A/C) based on the identity of residue 15.

To test this hypothesis, we designed a series of sixteen synthetic operators based on the consensus *E*. *coli* operator sequence [9]. We fixed the left half-site and altered only positions 14 and 15 of the right half-site by making all possible Watson-Crick base pair combinations at these sites (Fig 4A). We then created fluorescently labeled dsDNA probes of these operators and determined their LexA-operator dissociation rates using our fluorescence anisotropy assay. We found the dissociation rates spanned a wide range (1.9 s ≤ t_1/2_ ≤ 492 s) for this series of synthetic operator probes (Fig 5), thus validating our design hypothesis. Next, to construct the series of synthetic SOS promoters we sought, we used site-directed mutagenesis to engineer these same sixteen operators individually into the *recA* promoter GFP-reporter plasmid. We additionally chose operators from five different *E*. *coli* genes to engineer into the *recA* promoter to make a total of 22 reporter plasmids (including the native *recA* sequence) (Fig 4A). These added operators do not disrupt the -10 and -35 sites of the *recA* promoter, but add variability in the internal portions of the operator sequence that expanded the range of LexA-operator dissociation rates of this series to >3 logs. The series thus preserves the *recA* promoter structure but exploits targeted alterations in the SOS operator to span the entire range of native *E*. *coli* LexA-operator strengths.

To evaluate if our engineering disrupted promoter function or not, we measured the basal GFP-reporter activity of the plasmid series in Δ*lexA*, *lexA*^+^, and *lexA*^S119A^ cells in the absence of UV exposure (Fig 4C). If promoter function was unperturbed, then GFP activity should be similar between the plasmids in Δ*lexA* cells and differences in repression should only be apparent in *lexA*^+^ and *lexA*^S119A^ cells, where the repressor activity of LexA is expressed. As anticipated, we found that in Δ*lexA* cells, the promoter alterations had minimal impact on relative GFP signals (GPF/OD range < 0.5 logs) and exhibited no correlation with LexA-operator dissociation rate (r = 0.17, P = 0.45), whereas in *lexA*^+^ and *lexA*^S119A^ cells, we found relatively lower GFP signals over a wider range (GPF/OD range: 1.8 logs) which strongly correlated with LexA-operator dissociation rate (r = -0.95, P< 0.0001 for both strains). These results confirm that our promoter design did not significantly disrupt the RNA polymerase interaction and demonstrate the LexA-operator interaction can titrate the degree of basal transcription over a broad range. In this comparison, we also noted the data from the *lexA*^+^ and *lexA*^S119A^ strains were indistinguishable. This result suggests that basal cleavage of LexA does not contribute to basal transcription from SOS genes for the growth conditions used here, and that any loss of repressor is slow enough to be fully compensated for by new LexA synthesis.

### Analysis of operator residues 14 and 15 on LexA binding and promoter activity

Our systematic alteration of operator residues 14 and 15 to all sixteen possible Watson-Crick base pair combinations offered added insights into the LexA-DNA interaction and enabled us to infer the role of these residues in modulating promoter activity in cells. First, we analyzed the biochemically determined dissociation rates of LexA from the sixteen operator sequences by residue position (Fig 5A). At residue 15 of the operator DNA, A or C favored a stronger interaction (larger t_1/2_) over G or T. Inspection of the available crystal structure of the LexA-operator complex shows the interaction of A15 with LexA is stabilized through a single bridging water molecule that forms hydrogen bonds with A15 of the operator DNA and Asn41 of LexA [10]. The exocyclic N4 amine of C is also likely to support hydrogen bonding with Asn41 at this position, whereas the carbonyl oxygens of G and T would likely disrupt this interaction, thus explaining the structural basis for the discrimination we observe in our data. At position 14 of the operator DNA, we found that T formed a tighter complex than A, C, or G, with G forming the weakest complex. These findings were independent of the identity of residue 15. These results agree with the interpretation of Zhang, et al. that the methyl group of T interacts specifically with a hydrophobic pocket within LexA, thus stabilizing the LexA interaction [10]. Next, to understand if the biochemical data were predictive of the promoter behavior in live cells, we measured PA_peak_ values in live cells after a UV dose of 10 J/m^2^ for the sixteen reporter plasmids that harbor the same base pair substitutions. When PA_peak_ values were plotted against the dissociation rates (Fig 5B), we found a strong correlation (r = -0.99, P< 0.0001) between these data sets. Thus, the biochemical assay of LexA-operator dissociation rate determined *in vitro*, accurately predicts the promoter behavior in live cells for this system. We conclude that residue 15 plays a larger role in sequence discrimination than residue 14 and that both interactions significantly contribute to the differential promoter activation observed across the SOS gene network *in vivo*.

### Effect of DNA damage dose on synthetic *recA* promoters

Having established that PA_peak_ is governed by LexA-operator interactions at a fixed UV dose, we next aimed to examine how these interactions influence the threshold (ED_50_) and timing (t_peak_) of promoter activation. To do this, we acquired temporal promoter activity profiles over a range of UV doses for fourteen of the synthetic promoters, which spanned the entire range of LexA-operator dissociation rates (S7 Fig). Then, we analyzed these data using the same dose-response model and timing parameters as we applied to the native *E*. *coli* SOS promoters discussed above.

**Fig 6:**
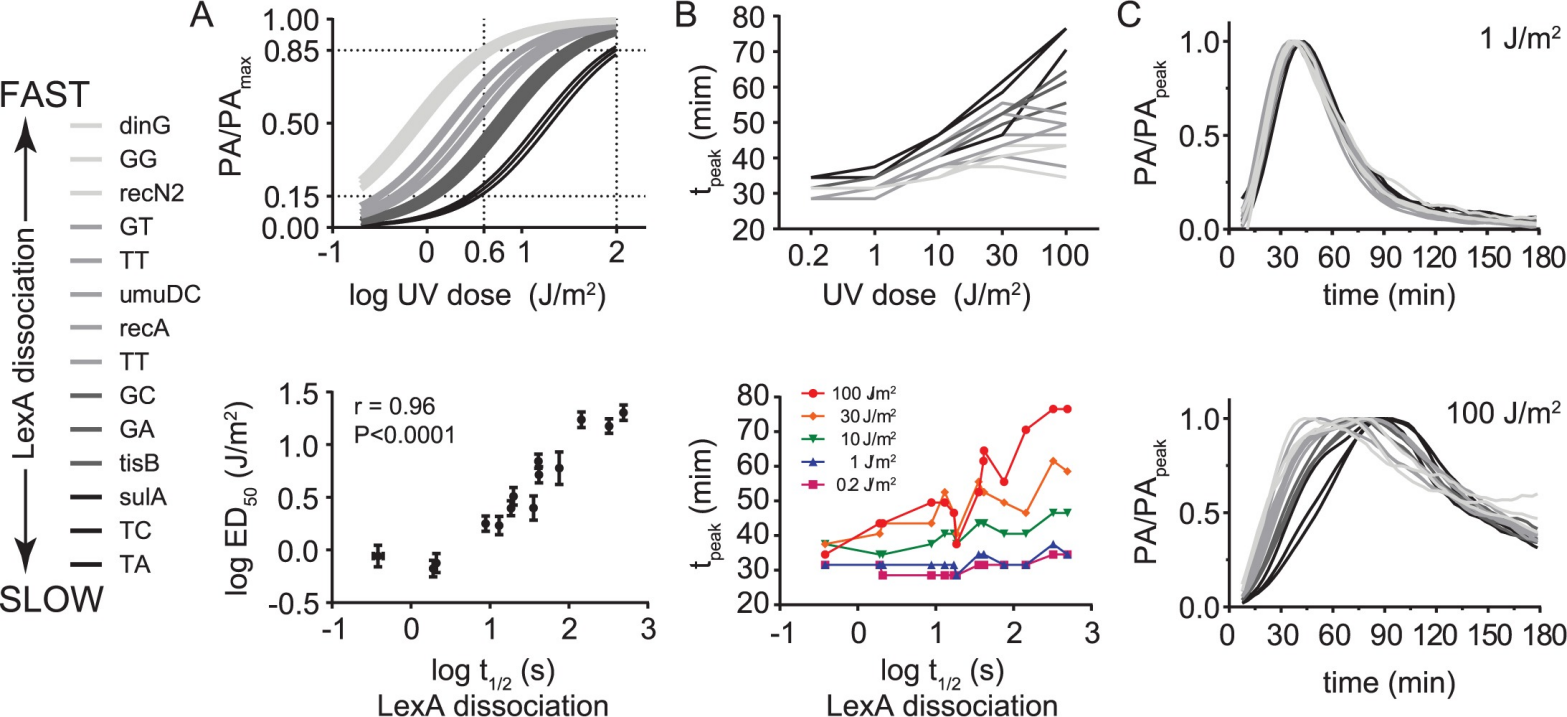
Effect of DNA damage dose on synthetic *recA* promoters. A. Top: Normalized dose-response curves for synthetic *recA* promoters. Bottom: Correlation between the UV-activation thresholds (ED_50_) derived from the dose-response model and LexA-operator dissociation rates (t_1/2_). B. Top: Plot of t_peak_ versus UV dose for synthetic *recA* promoters. Bottom: Plot of t_peak_ versus LexA-operator dissociation rate (t_1/2_) for each UV dose. Lines connecting data points from different UV doses (top) or t_1/2_ values (bottom) are shown for ease of visualization. C. Normalized promoter activity traces at UV doses of 1 J/m^2^ (top) and 100 J/m^2^ (bottom). Legend: Data lines with darker shading indicate slower LexA-operator dissociation rates (larger t_1/2_).

The PA_peak_ data fit well to the same dose-response model used above (R^2^ range = 0.93–0.99) allowing us to compare the fourteen dose-response curves and extract UV dose activation thresholds (ED_50_) for each promoter (S2 Table). While all promoters transitioned from being relatively inactive to near-maximally active over the dose range, at intermediate amounts of DNA damage a wide range of activation was observed (Fig 6A, top). To see if the LexA-operator interaction was responsible for this wide range, we plotted the ED_50_ values versus the LexA-operator dissociation half-life values (Fig 6A, bottom) and found a high dependency of this phenomenon on the LexA-operator dissociation rate (r = 0.96, P< 0.0001). We conclude that the strength of the LexA-operator interaction determines the promoter activation threshold (ED_50_) and thus enables the relative promoter activities in the network to change as a function of the amount of DNA damage incurred by the cell.

Next, we analyzed the relationship between the dose of DNA damage, the LexA-operator dissociation rate, and the timing of peak promoter activity. At the lowest dose of DNA damage, as with our observations using native promoters, we observed the t_peak_ values spanned a relative narrow range of 6 min (29–35 min). However, at higher doses of DNA damage we observed larger t_peak_ values for some promoters than for others, resulting in a significantly greater span of 43 min (34–77 min) in t_peak_ values at the highest dose (Fig 6B, top). To understand if these changes were a function of the LexA-operator dissociation rate, we plotted the t_peak_ values for each promoter versus the LexA-operator dissociation half-life values (t_1/2_) (Fig 6B, bottom) and found the dissociation rates of these promoters were highly predictive of t_peak_ values, particularly at higher UV doses (r = 0.90, P<0.0001 at 100 J/m^2^). Given that our synthetic promoter series isolates the LexA-operator interaction as the only variable between the various reporter plasmids, we conclude this interaction can control the timing of promoter activity, imposing peak promoter activity differences of greater than 40 minutes at high doses of DNA damage. To understand if these timing differences could be due to non-equilibrium dynamics of LexA binding kinetics at promoters, we plotted the normalized promoter activity traces together. If equilibrium dynamics prevail, then these traces are expected to be superimposable. However, we found the traces were only superimposable at lower doses of DNA damage (≤ 1 J/m^2^), whereas at higher doses of DNA damage, the promoters with slower LexA-operator dissociation rates were significantly right-shifted (Fig 6C). This latter finding is not consistent with an equilibrium model of LexA-operator dynamics (Fig 1).

### Evidence of non-equilibrium dynamics on the *E*. *coli* chromosome

**Fig 7:**
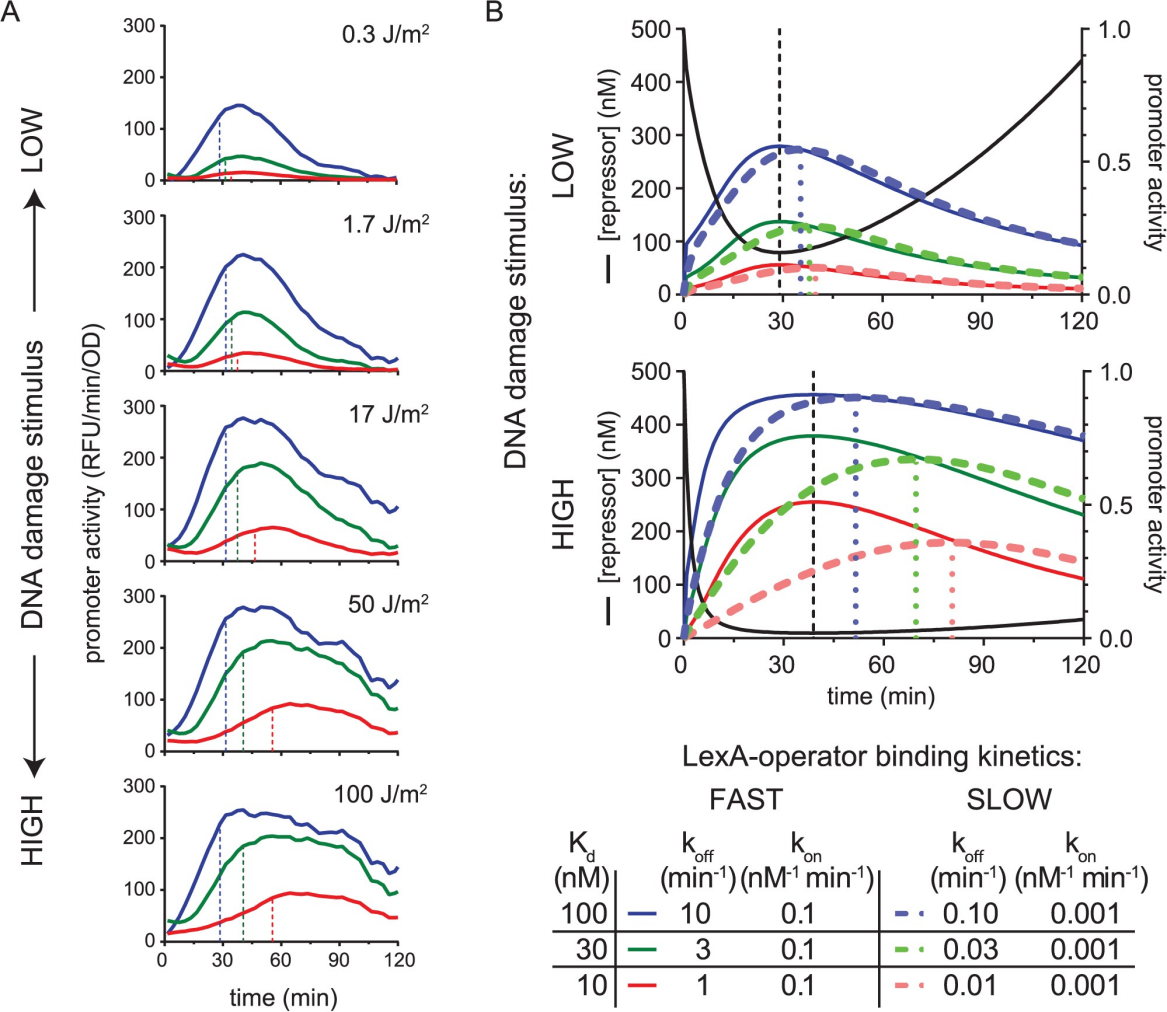
Chromosomal promoter activity data and a kinetic model of LexA occupancy at SOS promoters. A. Promoter activity kinetics of chromosomal promoters. MG1655 strains harboring chromosomal GFP-reporter cassettes with slow (red), intermediate (green), and fast (blue) LexA-operator dissociation kinetics were analyzed as above. These strains contain *recA* promoters with mutated LexA operators identical to those in Fig 4A and Fig 5. Strain MC0001 (red) contains the “TA” (consensus) LexA operator sequence, MC0002 (green) contains the “GA” sequence, and strain MC0003 (blue) contains the “GG” sequence. B. Kinetic model of LexA occupancy at SOS promoters. Modeled LexA depletion curves (black solid lines) are shown for a low (top) and high (bottom) UV dose. Colored solid and dashed lines indicate promoters with fast and slow LexA-operator binding kinetics, respectively. Values of modeled kinetic parameters are given in the figure legend, with ‘FAST’ kinetics allowing for thermodynamic equilibrium (as per the equilibrium model in Fig 1), while ‘SLOW’ kinetics create non-equilibrium dynamics. Black vertical dashed lines indicate the time of the LexA nadir. Colored vertical dashed lines indicate the time of peak promoter activity. Right axis: promoter activity ranges from zero to one, with zero meaning full LexA occupancy at the promoter and one meaning no LexA occupancy. The simulations show that accounting for non-equilibrium LexA-operator binding kinetics recapitulates the key features of the UV dose-dependent differences in timing observed in the experimental data: At high doses of DNA damage, promoters with slower LexA binding kinetics display peak activity at later times and more right-shifted temporal promoter activity plots.

To this point, our experiments utilized a plasmid-based GFP transcription-reporter system, however, there are many differences between plasmids and the *E*. *coli* chromosome which could potentially affect our results. For example, although the plasmid used above has a very low copy number (~5 copies/cell), the presence of additional LexA operators inside the cell might still alter the normal autoregulation at the *lexA* locus and skew our results. Additionally, the superhelical state, replication mechanism, and associated proteins are different between plasmids and the chromosome. Therefore, to understand if these differences could account for our results, we engineered three *E*. *coli* strains containing GFP-reporter cassettes inserted as a single copy at the *lacIZYI* locus of the chromosome, and repeated our measurements of promoter activity kinetics. These strains contain *recA* promoters with mutated LexA operators identical to those found in Fig 4A and Fig 5 and span the entire range of LexA-operator dissociation kinetics: Strain MC0001 contains the “TA” (consensus) LexA operator sequence (slow dissociation), MC0002 contains the “GA” sequence (intermediate dissociation), and strain MC0003 contains the “GG” sequence (fast dissociation). Analysis of these data replicated the key features described above in the plasmid-based system (Fig 7A). Specifically, at low UV doses, t_peak_ values were similar, whereas at high UV doses, t_peak_ values differed significantly, as a function of the LexA-operator dissociation rate. We conclude that non-equilibrium dynamics of the LexA-operator interaction also occurs on the chromosome.

### Kinetic model of LexA occupancy at SOS promoters

Given that our experimental results strongly suggested non-equilibrium dynamics, we sought a kinetic framework to model and test whether the combination of slow LexA binding kinetics at promoters and changing LexA concentrations could, in principle, explain the DNA damage dose-dependent effects on t_peak_ values in our data set. To do this, we first constructed a kinetic model for a generic repressor-promoter dissociation reaction using KinTek Explorer [26] software, then tested the model’s ability to recapitulate the key DNA damage dose-dependent features of our experimental data. We accomplished the latter part by subjecting the kinetic model to two different time-varying LexA repressor depletion curves using published immunoblot data of LexA depletion kinetics as a guide [13]. One curve represents a UV-dose of ~5 J/m^2^ and the other curve estimates the effect of a dose >20 J/m^2^. The strength of a kinetic framework is the ability to study the effect that reaction kinetics have on the formation of product, rather than the effect of equilibrium quantities. Thus, unlike models which assume thermodynamic equilibrium, a kinetic model enables the study of both equilibrium and non-equilibrium reaction conditions, by assigning ‘fast’ or ‘slow’ kinetic values, respectively, for the LexA-DNA binding reaction.

We used a simple dissociation reaction to model the interaction of a repressor, R, with its promoter, P:

R:P⇌R+P (1)

The rate constants for the dissociation and association reactions, k_off_ and k_on_, respectively, relate the concentrations of the reactants to the reaction rates:

$$Rate_{d}=\frac{-d\left[LexA:P\right]}{dt}=k_{off}\left[LexA:P\right]$$ (2)

$$Rate_{a}=\frac{d\left[LexA:P\right]}{dt}=k_{on}\left[LexA][P\right]$$ (3)

The equilibrium binding constant (K_d_) for the dissociation reaction is given by:

$$K_{d}=\frac{k_{off}}{k_{on}}$$ (4)

In this scheme, the promoter has two states: repressor occupied (R:P) and unoccupied (P). For our simple purpose of understanding the timing of promoter activity peaks, we assumed that the probability of making an RNA transcript is proportional to the fraction of promoter that is in the unoccupied state. Thus, in this model, promoter occupancy is being used as an approximation of promoter activity. To accomplish this, we fixed the total concentration of each promoter, *[P]*_*T*_ *= [P] + [LexA*:*P]*, to be constant. Then, we defined the activity of the promoter, *PA*, as being equal to the fraction of promoter that is unoccupied:

$$PA\left(t\right)=\frac{\left[P\right]\left(t\right)}{{\left[P\right]}_{T}}$$ (5)

Thus, *PA*_,_ has a range of values between zero and one, with zero representing no promoter activity (total repressor occupancy) and one representing maximal promoter activity (no repressor occupancy). We set promoters to have a concentration of 1 nM, since this is the approximate concentration of a single piece of promoter DNA in the volume of an *E*. *coli* cell. Repressor depletion curves started at 500 nM and, in the higher UV-dose we modeled, ranged as low as 10 nM. These values were chosen based on literature estimates of LexA concentrations in *E*. *coli* cells during the SOS response [13]. This model also assumes that operator bound form of LexA is resistant to RecA*-stimulated self-cleavage, which is also consistent with the literature [17, 21].

Using this kinetic model, we subjected six *in silico* promoters, each with a different set of kinetic constants (k_off_, k_on_), to the two different LexA depletions curves, representing either a low (~5 J/m^2^) or high (>20 J/m^2^) dose of DNA damage (Fig 7). We modeled one set of three promoters with fast binding kinetics and another set of three promoters with slow binding kinetics, with each set assigned the same K_d_ values of 10 nM, 30 nM, and 100 nM. To simulate fast kinetics, a relatively high k_on_ value of 0.1 nM^-1^min^-1^ was chosen for each promoter. To simulate slow kinetics, we chose a lower k_on_ value of 0.001 nM^-1^min^-1^. As expected for the promoters with fast kinetics, the simulation predicted the timing of peak activity for all the promoters to coincide with the timing of the nadir of LexA concentration (Fig 7B, Kinetics: FAST). Similarly, the K_d_ values, in conjunction with the repressor concentration, predicted the promoter occupancy at every time-point of the curve for these promoters. This reflects that, under these ‘FAST’ conditions, the repressor-promoter dissociation reaction is in thermodynamic equilibrium with falling and rising repressor levels. Of note, we found k_on_ values of ≥ 0.1 nM^-1^min^-1^ in this K_d_ range to reflect equilibrium conditions in this system for both the low and high stimulus conditions.

In contrast, the promoters with slow binding kinetics exhibited a different behavior (Fig 7B, Kinetics: SLOW), which replicated the key features or our experimental data. At the low dose of DNA damage, all three promoters displayed similar timing of peak promoter activity, over a range of just 5 min (35–40 min). However, at the high dose of DNA damage, the times of peak promoter activity occurred over a much wider range of 30 min (51–81 min). Interestingly, when we included a term in the model that permitted the operator-bound form of LexA to undergo RecA*-dependent self-cleavage at the same rate as free LexA, the timing differences at the high stimulus doses became negligible again (S8 Fig). This latter finding is consistent with data showing the LexA-operator complex is resistant to RecA*-induced LexA self-cleavage [17, 21] (see Discussion).

The above kinetic simulations revealed the requirements for peak promoter activity timing differences to emerge in a DNA damage dose-dependent manner. First, there must be a kinetic mismatch (i.e. a difference in relative time-scales) between the rate of LexA depletion/re-accumulation within the cell and the rate of LexA dissociation/association at the promoter, such that the latter becomes the rate-limiting step that dictates LexA occupancy at the promoter. Second, the dose of DNA damage must be sufficiently high to cause a long duration of low LexA levels in the cell. If these two requirements are met, then the timing of peak promoter activity will be significantly delayed compared to the time at which the LexA nadir occurred (Fig 7B, DNA damage dose: HIGH, Kinetics: SLOW). For example, in the latter half of the high DNA damage dose plots, promoter activity continues to increase for the promoters with slow kinetics even though repressor concentrations have begun rising at these later times. This paradoxical behavior can occur because the LexA binding kinetics remain out of equilibrium with the concentration of repressor at these times. Despite this disequilibrium, however, if LexA levels quickly re-accumulate (as is the case for low doses of DNA damage), then LexA occupancy will be rapidly re-established at promoters, thus mimicking the promoter activity pattern of the FAST exchange promoters (Fig 7B, DNA damage dose: LOW). In summary, the simulations show how LexA depletion curves with more prolonged low-levels of LexA can unmask a rate-limiting LexA binding step that determines the timing of peak promoter activity. Given that greater amounts of DNA damage are known to result in LexA depletion curves with more prolonged low-levels of LexA [13], we conclude that slow LexA binding kinetics at promoters can explain the dose-response relationship we observe for the timing of peak promoter activity.

## Discussion

Our findings demonstrate how a single transcription factor can be employed to modulate both the extent and timing of peak promoter activities across an entire gene network as a function of stimulus dose. Importantly, by engineering the entire range of LexA-operator strengths found in the *E*. *coli* chromosome into a single promoter template and studying a wide dose-range of DNA damage, we captured the full dynamic range at which the LexA-operator interaction modulates promoter activity in this organism. Interestingly, as the dose of DNA damage incurred by the cell increases, the network activation pattern changes not only in terms of relative gene induction, but also in terms of the relative timing of peak activation for each gene. Our experiments which isolated the effect of the LexA-operator interaction within the promoter, together with simulations using a simple kinetic model of promoter occupancy for LexA, show how sufficiently slow repressor binding kinetics at promoters can become rate limiting and determine the timing of peak promoter activity. This single molecular interaction, between repressor and operator, thus links the magnitude of an environmental stimulus to both the extent and timing of peak promoter activity. This finding also highlights the importance of using kinetic parameters, rather than the more commonly employed thermodynamic equilibrium parameters, to more accurately model biochemical pathways. However, since the kinetic parameters of the majority of gene regulation networks are unknown, our method of engineering a wide range of operator strengths into a common promoter template more practically serves as a diagnostic paradigm to test for non-equilibrium dynamics in other systems. To this point, a prior thermodynamic equilibrium model of LexA repression in the SOS network does not predict timing differences between SOS genes [15]. Our findings are particularly notable in that a kinetic model provides a mechanism by which a single transcriptional regulator, rather than cascades of transcription factors, can account for large timing differences of gene expression in a pathway.

Non-equilibrium dynamics of LexA at promoters are consistent with other features of the SOS response. First, the LexA-operator complex is resistant to RecA*-stimulated self-cleavage [17, 21] and, second, RecA is sequestered to discrete foci in cells after DNA damage [27, 28] and thus may be limited in its ability to diffuse to sites in the genome where LexA is bound to operator DNA. Both of these mechanisms suggest the DNA-free form of LexA is depleted preferentially over the DNA-bound form inside cells. This increases the relevance of LexA binding kinetics at promoters since LexA must dissociate before it can be cleaved by RecA. Thus, it is plausible that the preferential depletion of the DNA-free form of LexA is a requirement for large timing differences to emerge within the network, which is consistent with our simulations of operator occupancy.

This study also uncovered that the relationship between the dose of DNA damage and the timing of peak promoter activity in the SOS gene network occurs on a larger dose and time scale than previously recognized. At low doses of UV-induced DNA damage the timing is actually identical between genes. Differences begin to emerge at doses >1 J/m^2^ and at 100 J/m^2^ the differences can be nearly 60 min between some genes, which is much larger than formerly appreciated. This relationship had been obscured since past studies have been limited to a relatively narrow dose-range of UV-induced damage, lacked the temporal resolution required to assign peak activity times, or studied relatively few genes. For example, microarrays were used to measure the increase in mRNA transcripts for the entire *E*. *coli* transcriptome at 5, 10, 20, 40, and 60 min after a single UV dose of 40 J/m^2^ [9]. This important study helped define all the genes in the SOS regulon, but did not uncover significant timing differences between genes. This apparent disparity may be due to the methodology employed, as microarrays only measure the relative abundance of transcripts and not the *rate* of transcription, and the number of time points analyzed in the study was likely still too low to reliably infer the rates. Furthermore, microarrays can be prone to signal saturation due to excess RNA, which may have obscured linear changes in RNA abundance over the time course of the experiment. Ronen, et al. overcame these technical barriers by studying seven SOS gene promoters at doses of 5 and 20 J/m^2^ using the GFP-reporter plasmid system, thus enabling precision quantification of promoter activity through time [15]. Although timing differences were noted, a correlation between UV dose and timing was not investigated, probably due to the relatively narrow dose-range and lower number of genes studied. Thus, our study builds upon these previous reports, but, by studying fourteen SOS promoters over a wider dose-range of DNA damage with the high temporal resolution GFP-reporter system, our more comprehensive evaluation both helps to confirm the existence of timing differences in the SOS pathway and reveal the full scope of the relationship between UV dose and timing. We also found that studying a wide dose-range of DNA damage with several intermediate doses was important for accurately assigning promoter activation threshold values (ED_50_) to genes in our dose-response model of peak promoter activity. This may explain the differences in the rank-ordering of our ED_50_ values compared to a prior study which parameterized activation thresholds of SOS genes using only two different UV doses over a narrower dose-range [15].

Furthermore, our kinetic model of LexA occupancy yields a comprehensive view of transcriptional timing within the SOS network. Our results generally confirm the inferred temporal ordering of genes from past studies, showing a transition from nucleotide excision and recombinational repair to error-prone repair and the arrest of cell division [16], but now shows this ordered progression of transcription only manifests at high amounts of DNA damage. Of note, at the lowest dose of DNA damage, the promoter activity of *umuD* was barely detectable, therefore, it is possible that some genes are only transcribed in physiologically relevant quantities with higher amounts of DNA damage, thus manifesting only at late times. Several studies have uncovered the importance of LexA-independent modes of transcriptional regulation for the timing of expression of individual SOS genes, such as catabolite repression of the plasmid-borne colicin E1 gene through the cAMP-CRP complex [19] or alternative sigma-factor utilization of *dinB* through RpoS (σ^32^) [18]. Our data also suggest alternative sigma-factor utilization for *ruvA*, *sbmC*, and *ybfE*. Thus, we favor an overall model where the LexA binding kinetics at each SOS promoter dictates a baseline for timing as a function of the amount of DNA damage present and that additional mechanisms serve to further fine-tune that timing in response to certain environmental conditions. One limitation of our study is that we only manipulated a single promoter template (*recA*), but additional factors found in other promoters may also be important for timing, such as the number of LexA operators and their positions relative to the transcription start site or the inherent strength of each promoter [11]. In future studies, an analogous approach using different SOS promoter templates to isolate these other features could quantify their significance.

Another limitation is that we studied cloned promoter fragments out of the context of their native loci. Although the fragments studied here capture the entire intergenic region between the adjacent open reading frames (S1 Fig), chromosomal factors which influence transcription over longer distances could have been missed. However, our study shows that the kinetic mechanism described above is directly attributable to the LexA-operator interaction at the promoter, and the findings hold with plasmid-based or chromosomal reporters. Therefore, if factors exist which operate over longer stretches of the chromosome, they would only serve to interfere with the disequilibrium mechanism described above.

Non-equilibrium dynamics of LexA binding at SOS promoters is particularly intriguing in the context of two different findings from studies of promoter activity in single cells using fluorescence microscopy. First, in one study, the promoter activity of three SOS genes was examined through time after UV-induced DNA damage, which uncovered an oscillatory behavior of promoter activity in these genes [29]. The *recA* promoter was studied in greater detail over a UV dose-range of 10–50 J/m^2^. After a UV stimulus, the promoter responded with three successive ‘pulses’ of activity, with larger UV doses resulting in higher amplitudes of the subsequent pulses. The mechanism underlying this phenomenon remains to be experimentally elucidated, although different mathematical models of the SOS feedback circuit can produce oscillatory behavior [30–32]. Our finding that slow LexA exchange kinetics at the *recA* promoter can delay the aggregate peak timing in a population of cells in a UV-dose dependent manner suggests this same mechanism, in an individual cell, is responsible for adjusting the relative amplitudes of the promoter activity pulses. Second, spontaneous activation of SOS gene promoters has been documented in subpopulations of cells [33, 34], suggesting activation of the SOS response could be due to a low rate of spontaneous DNA damage in the population. However, by studying two different SOS genes (*lexA* and *cka*) simultaneously in the same cell with different colored fluorescent reporters, examples were also found where only *cka* was spontaneously activated [34]. This unlinked behavior is more difficult to attribute to spontaneous SOS activation and therefore likely represents stochastic gene expression. Our finding of slow LexA binding kinetics at some SOS genes provides one possible explanation for this behavior: if re-association of LexA is kinetically slow after LexA dissociates from a promoter, then the time window where the promoter is unoccupied may permit RNAP to initiate transcription. Future studies simultaneously monitoring multiple synthetic SOS genes, which share a common promoter template but differ in LexA-promoter binding kinetics, may be able to provide evidence for this hypothesis.

## Materials and methods

### Bacterial strains, plasmids, and oligonucleotides

An *E*. *coli* K12 MG1655 Δ*sulA*::*FRT* strain (SAMP04-*lexA*^WT^) was used for experiments and related strains (SAMP04 and SAMP04-*lexA*^S119A^) were used for controls. Construction of these strains has been described previously [35]. SAMP04 is Δ*lexA* and the *lexA*^WT^ and *lexA*^S119A^ variant alleles are in the native context. *sulA*^*+*^ strains are not viable in the Δ*lexA* background due to constitutive inhibition of cell division, therefore the Δ*sulA* background permits parallel comparison in the control strains.

The lambda red recombineering system [36] was used to construct *E*. *coli* strains with chromosomally encoded GFP-reporter cassettes. Briefly, a wild type *E*. *coli* K12 MG1655 strain harboring the temperature sensitive plasmid pKD46 (MG1655/pKD46), which encodes for the lambda red *gam*, *bet*, and *exo* genes, was used for recombineering. The DNA used for recombination with this strain was amplified from pUA66-recA derivatives by PCR using primers containing ~50 bp of homology to the *lacI* and *lacA* genes (S3 Table). These primers amplify a 2.1 kb region of the plasmid that contains Kan^R^, the cloned *recA* promoter region, and *gfp-mut2* (S1 Fig) to make a Kan^R^-recA_p_-*gfp* cassette suitable or recombination at the *lacIZYA* locus. To remove the template plasmid DNA, PCR reactions were treated with DpnI, subjected to electrophoresis, and the desired product band was gel-extracted. The DNA was then concentrated by ethanol precipitation and electroporated into MG1655/pKD46 cells. Recombinants were screened for both kanamycin resistance and loss of *lacZ* activity and then verified using PCR primers to detect replacement of the *lacIZYA* locus with the Kan^R^-recA_p_-*gfp* cassette. These strains contain *recA* promoters with mutated LexA operators identical to those in Fig 4A and are designated as follows: Strain MC0001 contains the “TA” (consensus) LexA operator sequence, MC0002 contains the “GA” sequence, and strain MC0003 contains the “GG” sequence. For each strain, two independent clones were selected for further analysis.

GFP reporter plasmids were obtained from the GE Dharmacon *E*. *coli* promoter collection, with complete sequences obtainable as described in S1 Fig [22]. Site-directed mutagenesis was carried out on the *recA* reporter plasmid by PCR using primers containing the desired mutations (S3 Table). Mutations were confirmed by DNA sequencing. Synthetic DNA oligonucleotides were purchased from Integrated DNA Technologies.

### Measurement and analysis of temporal promoter activity

Bacteria transformed with GFP-reporter plasmids were cultured in defined media containing 1x M9 salts (Sigma M6030), 0.4% glucose, 2 mM MgSO_4_, 0.1 mM CaCl_2_, 0.05% casamino acids, and 30 μg/ml of kanamycin. To start an experiment, 2–3 mL of media was inoculated with a 1:100 dilution of overnight culture and incubated with shaking at 37 ^o^C and 225 rpm. After 2–2.5 hours of incubation, cultures achieved an optical density at 595 nm (OD595) of ~0.3 and 100 μL aliquots were dispensed into a 96-well clear bottom black plate (Corning, CoStar #3603). The typical assay plate contained seven rows containing bacterial culture, with each row representing a different GFP-reporter plasmid, and a final row containing blank wells (media only). After plate dispensing was complete, a germicidal lamp (UVP, LLC) set to 254 nm (fluence rate = 1350 μW∙cm^-2^ at 3 inches) was used to irradiate the cells and the UV dose was adjusted by varying the distance and time of exposure. To achieve different UV doses on the same plate, aluminum foil was used to cover all but two columns of the assay plate at a time. This allowed for six different UV exposure conditions on a single plate (No UV, 0.2, 1, 10, 30, and 100 J/m^2^), with each UV condition assayed in duplicate for every GFP reporter plasmid on the plate. After UV exposure, GFP fluorescence intensity (RFU) and absorbance at 595 nm (OD) were acquired every 3 minutes on a Tecan Infinite F200 Pro multifunction plate reader in kinetic mode for at least 150 minutes. Temperature was maintained at 37 ^o^C and cultures were agitated before every data acquisition cycle. Each experiment was repeated on a separate day, therefore, each curve consists of at least 50 time points, with each time point assayed in quadruplicate across two separate days. For experiments using strains harboring a chromosomal reporter cassette (MC0001, MC0003, and MC0003) kanamycin was omitted from the culture media and the promoter activity data from two independent recombinants were averaged.

Promoter activity was calculated as previously described [15]. GraphPad Prism was used to take the first-derivative of the raw ‘RFU versus time’ data and apply a smoothing function to reduce noise within the data set and improve precision. The smoothing function replaced each time-point with the value obtained by averaging the time-point with its two neighbors, and replicates within the same plate were also averaged in this process. Final OD values were calculated by subtracting data from blank wells. To control for cell growth, the first-derivative values were normalized to cell density by dividing by the OD values. Finally, background signal was removed by subtracting the ‘No UV’ condition from each data set. The resulting values for promoter activity (PA), with units of RFU/min/OD, were used for analysis. PA_peak_ was defined as the highest promoter activity value in the trace. Promoters were parameterized by fitting the PA_peak_ values to a three-parameter dose-response model using non-linear regression with the following equation:

$$PA_{peak}=\frac{PA_{max}}{1+10^{\left(LogED_{50}-X\right)}}$$ (6)

where X = the log of the UV dose applied to the cells.

### LexA overexpression and purification

The *E*. *coli lexA* gene was PCR amplified using primers containing the NdeI or XhoI restriction sites and a 5’-hexahistidine sequence (S3 Table), then cloned into a pET41 plasmid vector. To overexpress LexA, *E*. *coli* BL21(DE3) cells harboring the plasmid were grown to mid-log phase at 37 ^o^C, induced with 1 mM isopropyl β-D-1-thiogalactopyranoside, and harvested three hours after induction. To purify LexA, the cells were resuspended in lysis buffer (20 mM sodium phosphate, 500 mM sodium chloride, pH 6.6), sonicated at 4 ^o^C, and the resulting lysate was clarified by centrifugation and supplemented with 10 mM imidazole. Next, the supernatant was incubated with HisPur-Cobalt resin (Thermo-Fisher Scientific) and the protein-bound resin was washed with lysis buffer containing 20 mM imidazole, and then LexA was eluted from a column with lysis buffer containing 250 mM imidazole. Elution fractions were combined and dialyzed into gel-filtration buffer (10 mM PIPES, 150 mM sodium chloride, pH 6.6). The dialyzed sample was then injected onto a 16/600 200 pg gel-filtration column and fractions were analyzed by SDS-PAGE. LexA undergoes detectable autoproteolysis during overexpression and purification, however, the gel-filtration column efficiently separated full-length LexA from the contaminating LexA cleavage products. Fractions containing >99% full-length protein were combined and aliquots were stored at -80 ^o^C. Protein concentration was determined by Bradford assay.

### Determination of LexA-operator dissociation rates

Fluorescein amidite (FAM)-labeled double-stranded DNA (dsDNA) probes containing LexA operator sequences were constructed from synthetic oligonucleotides (S3 Table). First, the 3’-end of one DNA strand was labeled using 5-propargylamino-ddUTP-5-FAM (ddUTP-FAM) (Jena Bioscience) and terminal transferase (New England Biolabs) and the unincorporated label was removed using an oligonucleotide clean-up kit (Zymo). We estimated the labeling efficiency for FAM incorporation to be ~80% by UV-vis absorbance, using the ε_260_ for each oligonucleotide and ε_495_ = 75,000 M^-1^cm^-1^ for FAM. Then, the FAM-labeled strand was added to 1.5x molar excess of its unlabeled reverse complement DNA strand in a solution containing 70 mM Tris-HCl (pH 7.6), 10 mM MgCl_2_, and 100 mM NaCl and the two strands were annealed by heating to 95 ^o^C and cooling to 25 ^o^C over 140 min using a thermocycler.

LexA-operator dissociation rates were determined using a stopped-flow apparatus (KinTek) equipped for fluorescence anisotropy detection. First, LexA-DNA complexes were formed by incubating 400 nM LexA with 100 nM FAM-labeled dsDNA. Addition of LexA to FAM-labeled operators resulted in an increase in anisotropy. As expected, no change was observed using FAM-labeled scram operators (S3 Table). Then, the stopped-flow instrument was used to rapidly mix 20 μL of the LexA-DNA complex solution with an equal volume of a 2 μM solution of unlabeled DNA, which contained the consensus LexA operator sequence (S3 Table). After mixing, the decay of the fluorescence anisotropy signal was recorded through time for at least five half-lives. Prior to mixing, both solutions contained 70 mM Tris-HCl (pH 7.6), 10 mM MgCl_2_, 150 mM NaCl, 100 μg/mL bovine serum albumin, and 10 μg/mL sonicated salmon sperm DNA. Experiments were performed at room temperature. No anisotropy decay resulted from mixing scram operator DNA (S3 Table) with LexA-DNA complexes. Also, increasing the concentration of unlabeled operator DNA did not significantly alter the decay kinetics. To obtain kinetic parameters (t_1/2_, k_off_), data from three to twelve replicate experiments were combined and analyzed by non-linear regression using a simple exponential decay model in GraphPad Prism. Association rates were measured using the same reaction conditions and experimental setup, except that the stopped-flow apparatus was used to mix a solution containing the labeled operator DNA with a solution containing LexA. Also, association reactions were performed at 4 ^o^C instead of at room temperature, as the rate of the room temperature reactions exceeded the sampling rate of the instrument. The rate constant for the association reaction was estimated with nonlinear regression assuming pseudo-first order kinetics, where k’_on_ = k_on_[LexA]_o_.

### Determination of LexA-operator equilibrium binding constants

Electromobility shift assays were used to measure LexA-operator equilibrium binding to the same dsDNA probes used above for the dissociation assay (S3 Table), however, probes were labeled with ^32^P to facilitate detection of low concentrations of DNA. The 5’-end of one strand of the probe was ^32^P-labeled using γ-^32^P-ATP and T4 polynucleotide kinase (New England Biolabs) and the unincorporated γ-^32^P-ATP was removed using a G50 spin gel-filtration column (GE Healthcare). LexA-DNA binding reactions contained 10 pM ^32^P-labeled dsDNA probe. Each probe was studied over nine different LexA concentrations ranging from 0.2–1000 nM. Binding reactions were incubated at room temperature for 5–10 min in a solution containing 70 mM Tris-HCl, 2 mM PIPES (pH 7.5), 10 mM MgCl_2_, 200 mM NaCl, 100 μg/ml bovine serum albumin, 1 μg/mL sonicated salmon sperm DNA, 5% glycerol, and 0.006% bromophenol blue. Using our fluorescence polarization LexA-DNA binding assay, we found the binding reactions equilibrated in < 30 sec. Products of the reaction were separated by native-gel electrophoresis using 6% polyacrylamide mini-gels cast in 0.5x TBE buffer. Samples were applied to gels with current running. Electrophoresis was carried out at 4°C in 0.5x TBE buffer at ~10 V/cm. Each binding reaction was repeated at least twice. DNA bands were visualized by phosphorimaging using a Typhoon instrument and quantified using QuanityOne (BioRad). We observed one band corresponding to free probe (F) and one shifted band corresponding to the LexA-DNA complex (B). The fraction of bound probe (f_B_) for each reaction was calculated using f_B_ = B/(B+F). To extract apparent K_d_ values, data were fit to a one-site specific binding model using non-linear regression in GraphPad Prism.

### Statistical correlations

GraphPad Prism was used to test for rank-order statistical correlations using the non-parametric Spearman correlation coefficient, unless otherwise indicated. All reported P-values are two-tailed.

### Modeling of promoter activity

KinTek Explorer software was used to derive a kinetic model of repressor occupancy and repressor depletion curves [26]. The software uses numerical integration to simulate complex reaction schemes. The complete list of reaction schemes, rate constants, and starting concentrations, as well as a description of the LexA depletion model are provided in S4 Table.

## Supporting information

S1 Fig Reporter plasmids and control experiments. A. GFP reporter plasmids containing the cloned promoter regions were developed by Zaslaver, et al. at the Weizmann Institute of Science [22] and were obtained from the commercially available “*E*. *coli* promoter collection” (Dharmacon). The cloned promoter region for each plasmid contains the entire intergenic region between the open reading frame of the gene of interest (ORF2) and the open reading frame of the upstream gene (ORF1) plus about 50–150 bp into each flanking coding region. The DNA sequence of the cloning vector pUA66 can be found at the following URL: http://www.weizmann.ac.il/mcb/UriAlon/zaslaver-et-al-2006-nature-methods-2006. The complete list and chromosome location of cloned regions can be found at the following URL: http://dharmacon.gelifesciences.com/cdnas-and-orfs/non-mammalian-cdnas-and-orfs/e-coli/e-coli-promoter-collection/. B. Promoter activity (PA) after UV exposure requires LexA cleavage and is specific for SOS promoters. Activity from the GFP-reporter plasmid containing the SOS *recA* promoter transformed into cells expressing functional LexA (*lexA*^+^) (green) is dependent on UV exposure. Dashed lines indicate no UV exposure and solid lines indicate a UV dose of 10 J/m^2^. No activity is observed in a bacterial strain which encodes a LexA-variant incapable of self-cleavage (*lexA*^S119A^) (purple). No activity is observed for the non-SOS *lac* promoter (black).
(EPS)

S2 Fig Normalized temporal promoter activity traces arranged by UV dose. Promoter activity traces for all 14 promoters and UV doses (indicated to left of each plot) studied are shown. To facilitate comparison of temporal (x-axis) variation between traces, the promoter activity (PA) values of each trace were normalized by dividing by the highest value in that trace (PA_peak_) yielding normalized traces which all range in value from zero to one. The majority of the promoters are shown in grey-scale, with the exception of red (*sbmC*), blue (*ruvA*), and purple (*ybfE*) lines that indicate promoters with possible alternative sigma-factor regulation. ‘Early’ promoters are shown as light grey lines (*ssb*, *uvrD*, *lexA*, *ftsK*, and *dinG*), ‘middle’ promoters are shown as dark grey lines (*recA*, *recN*, *polB*, and *dinB*), and ‘late’ promoters are shown as black lines (*sulA* and *umuD*). Early, middle, and late designations are based on t_peak_ values and are the same as those from Fig 2B. Vertical dashes and horizontal lines above each plot indicate t_peak_ values and ranges, respectively. Data for *umuD*, *sbmC*, *and ybfE* were not included in the 0.2 J/m^2^ plot due to low signal-to-noise.
(EPS)

S3 Fig Temporal promoter activity traces of *E*. *coli* SOS promoters. Promoter activity traces are shown for each promoter in the format of Fig 2A. The data for *uvrD* and *sulA* are repeated here for ease of comparison.
(EPS)

S4 Fig Dose-response model of peak promoter activity. A. Dose-response model of PA_peak_ vs. UV dose for representative genes. Ticks on the right y-axis indicate estimated values for the maximal activity of the completely de-repressed promoter (PA_max_) as given by the top-asymptote value of the best-fit curve. Ticks on the x-axis indicate values for the UV dose which yields half-maximal promoter activity in the model (ED_50_). B. Correlation between basal GFP levels, measured in *lexA*^+^ or Δ*lexA* cells, and the PA_max_ values obtained from the dose-response model. The line of best fit is shown for the Δ*lexA* data set. Error bars are shown for PA_max_ values and represent the standard error derived from non-linear regression. When not shown, error bars are encompassed within the symbol.
(EPS)

S5 Fig Dissociation rates of tagged and tagless LexA. A. SDS-PAGE analysis of thrombin cleavage reactions. The purified LexA protein used for biochemical experiments (H_6_-LexA) contains an N-terminal hexahistidine tag (H_6_) that is removable by specific thrombin cleavage to yield a LexA protein with only three additional non-native amino acids appended to the N-terminus (LexA). The amino acid sequence of the tag is MGH_6_RRASQGLVPR/GSH-LexA, where H_6_ indicates the hexahistidine motif and the underlined portion and forward slash represent the thrombin recognition sequence and cleavage site, respectively. H_6_-LexA was incubated either without (-) or with (+) thrombin-sepharose beads. Beads were removed by centrifugation and proteins were dialyzed. Reaction products were separated by electrophoresis on a 15% SDS-PAGE gel, then visualized by staining with Coomassie brilliant blue dye. The analysis shows complete removal of the tag after incubation with thrombin. The faint bands are the C-terminal domain (CTD) and tagged N-terminal domain (H_6_-NTD) products of LexA autocleavage, along with the corresponding thrombin digestion product (NTD). B. Dissociation rates of tagged and tagless LexA. The fluorescence anisotropy assay was used to compare the dissociation rates of tagged (solid lines) and tagless (dotted lines) LexA from the consensus sequence operator probe (red) and the *sulA* operator probe (blue). Fluorescently labeled operator probes, reaction conditions, and analysis are as described in the main text, however, this experiment was performed in a 384-well plate using the fluorescence polarization mode of a Tecan Infinite F200 Pro multifunction plate reader instead of a stopped-flow instrument. Individual data points represent the average value of 4–6 independent replicates and curves represent the line of best fit from non-linear regression. The analysis shows that removal of the N-terminal tag results in slightly faster dissociation for both operator probes, but that the relative ratio of rates between operator probes are indistinguishable.
(EPS)

S6 Fig Plot of LexA-operator association rate constants versus dissociation rate constants. Plot of the pseudo-first order association rate constant, k’_on_, versus the dissociation rate constant, k_off_, for 16 LexA operator probes. Rate constants were determined using the FAM-labeled operator probes from Fig 5 and the fluorescence anisotropy assay (see Materials and Methods).
(EPS)

S7 Fig Temporal promoter activity traces of synthetic SOS promoters. Promoter activity traces are shown for each of the synthetic SOS promoters in the format of Fig 2A.
(EPS)

S8 Fig Effect of RecA*-mediated cleavage of the operator-bound form of LexA on the kinetic model of LexA occupancy of SOS promoters. Data are shown simulating a high DNA damage dose, as in Fig 7B, but here only three promoters are modeled, all with SLOW LexA-operator binding kinetics. Colors indicate equilibrium affinities. Traces are shown for a model where RecA*-mediated cleavage of the operator-bound form of LexA is permitted (solid lines) and a model where cleavage of the operator-bound form of LexA is prohibited (dotted lines). The former was accomplished by adding the reaction schemes, AB + PR ⇌ ABPR and ABPR ⇌ AB+ P, to the model and setting the rate constants equal to that of the free LexA cleavage reaction (see S4 Table, schemes 3 and 4). The simulation shows that permitting the operator-bound form of LexA to be cleaved reduces the timing differences between promoters.
(EPS)

S1 Table *E*. *coli* SOS promoter parameters. Errors reported for t_1/2_ values represent the largest tail of the 95% confidence interval derived from non-linear regression. Errors for ED_50_ and PA_max_ values represent the standard error derived from non-linear regression. The error reported for t_peak_ values is a conservative estimate based on the 3.0 min sampling period of the instrument. For promoters with more than one operator site, the given value is the largest t_1/2_ value of all the operators for that promoter. The number and DNA sequence of operators for each promoter is derived from Courcelle, et al. [9] and their position relative to the transcription start site is derived from the EcoCyc database [37]. Values for the position of the operator within the promoter refer to the bp location of the middle of the operator based on the convention that the transcription start site is +1.
(EPS)

S2 Table Synthetic SOS promoter parameters. Errors reported for t_1/2_ values represent the largest tail of the 95% confidence interval derived from non-linear regression. Errors for ED_50_ and PA_max_ values represent the standard error derived from non-linear regression.
(EPS)

S3 Table Oligonucleotides used in this study. A. DNA oligonucleotides used to construct dsDNA binding probes for the LexA-operator dissociation assay. Probes were constructed by 3’-labeling the 30-mer shown with ddUTP-FAM to obtain a 31-mer. The labeled strand was annealed with its 31-mer reverse complement. The 44-mer probes were not labeled, but used as the source of excess operator DNA for LexA-operator dissociation rate measurements. Scram sequences have the highly conserved CTG-motifs in reverse orientation, which ablates all detectable binding activity with LexA. B. DNA oligonucleotide primers used in this study. Top: Primers used for site-directed mutagenesis. Primer-directed mutagenesis was carried out in a PCR reaction using the GFP-reporter plasmid indicated in the table as the template DNA. Primer names refer to the desired product of mutagenesis. Only one strand of the primer pair is shown (in all cases, the other strand is the reverse complement). Note that *ysdAB* is synonymous with *tisB*. Bottom: GFPint_R is the primer used for DNA sequencing to confirm mutations. H6PKA_F and CtermStop_R are the primers used to amplify *lexA* and clone into the pET expression vector (see ‘Overexpression and purification of LexA’). lacI_pUA66_kan and lacA_pUA66_gfp are the primers used to amplify the Kan^R^-recA_p_-gfp cassette from pUA66 derivatives for lambda red recombineering. lac_F, lac_R, and lac_Rm are the primers used to confirm the lambda red mediated recombination.
(EPS)

S4 Table KinTek Explorer reaction schemes and parameter values. **‘**Repressor depletion’ curves mimicking LexA levels after a UV exposure of ~5 J/m^2^ (Stimulus: LOW) and >20 J/m^2^ (Stimulus: HIGH) were modeled using a combination of six simple reaction schemes. k+ and k- refer to the forward and reverse reaction rate constants (k_off_ and k_on_) for each scheme. Units for k+ (k_off_) are in min^-1^. Units for k- (k_on_) are in nM^-1^·min^-1^. Reaction schemes 1 and 2 enable a transient accumulation of species AB, which, by way of reaction schemes 3 and 4, functions to degrade the repressor, R. Reaction schemes 5 and 6 enable negative auto-feedback of new repressor synthesis and were required to achieve the rapid re-accumulation of repressor observed in the literature [13]. Although the choice of reaction schemes that result in repressor depletion curves which mimic literature observations for LexA levels (as shown in Fig 7) are arbitrary, the schemes chosen here do parallel SOS regulatory structure conceptually. The first four schemes model the formation of RecA* (AB) from RecA (A) and ssDNA (B) (scheme 1), the inactivation of RecA* (scheme 2), the formation of a LexA-RecA* complex (ABR) (scheme 3), and the RecA*-induced cleavage of LexA (R) (scheme 4). Scheme 6 models new LexA (R) synthesis by the *lexA* promoter (D) and scheme 5 makes LexA synthesis dependent on the LexA (R) occupancy state at the promoter (D), thus mimicking the known negative autoregulation of *lexA* transcription [38]. ‘Promoter occupancy’ was modeled using a simple promoter-repressor dissociation reaction (see Results). Mixing step values indicate the starting concentration (nM) of each species at the beginning of the simulation (t = 0).
(EPS)
